# Comment on “Differential Effects of the Temporal and Spatial Distribution of Audiovisual Stimuli on Cross‐Modal Spatial Recalibration”

**DOI:** 10.1111/ejn.15001

**Published:** 2020-10-27

**Authors:** Jean Vroomen, Jeroen J. Stekelenburg

**Affiliations:** ^1^ Department of Cognitive Neuropsychology Tilburg University Tilburg the Netherlands

## Abstract

Bruns et al. (2020) provide new research that suggests that the ventriloquism after‐effect (VAE: an enduring shift of the perceived location of a sound toward a previously seen visual stimulus) and multisensory enhancement (ME: an improvement in the precision of sound localization) may dissociate depending on the rate at which exposure stimuli are presented. They reported that the VAE, but not the ME, was diminished when exposure stimuli were presented at 10 Hz rather than at 2 Hz. To the authors, this suggested that different neural structures underlie the VAE and ME. In our view, however, this needs to be tested more extensively because alternative and simpler explanations have not yet been checked.

The brain tries to minimize sensory ambiguity and uncertainty by combining incoming signals into a unified percept. A remarkable example of this is the ventriloquist effect where the apparent location of a sound is attracted toward a slightly displaced visual stimulus like a flash that changes in synchrony with that sound. Not only is there a shift or even fusion of the perceived location of the sound toward the visual stimulus (the ventriloquist effect, VE) but there is also perceptual learning that is observable as an aftereffect, when sounds are presented later in isolation. This perceptual learning can manifest itself as an enduring bias or shift of unimodal of sound localization toward the previously seen visual stimulus (the ventriloquism after‐effect, VAE), or as a reduction in variance/ improvement in the precision of sound localization (multisensory enhancement, ME). The mechanisms supporting these capacities have remained somewhat of a puzzle, but new research (Bruns et al., 2020) suggests that the VAE and the ME, these two signs of recalibration that shift sound localization and reduce variance, may dissociate as data suggest that the VAE can falter while the ME remains intact.

Bruns et al. (2020) used a pretest‐exposure‐posttest design where participants pointed during pre‐ and posttests toward the apparent location of short tones emanating from one of six speakers (spanning −22° to +22°). During the intervening exposure phase, these tones were accompanied by synchronized lights that were either congruent with the location of the sound (0° disparity), or displaced by 13.5° to the right. The audiovisual exposure stimuli were presented for 5 min (600 stimuli in total) at either a constant rate of 2 Hz (low‐frequency stimulation), or at a much higher frequency of 10 Hz with a 9 s pause in between these audiovisual stimulus bursts. The VAE was then calculated by subtracting in the displaced conditions the pointing responses in pretest from posttest. The ME was calculated in congruent conditions as the reduction in absolute localization errors between pretest and posttest. The results showed a rightward shift of ~4.7° (the VAE) after low‐frequency stimulation, but this VAE was significantly reduced after high‐frequency stimulation (only a ~1.7° rightward shift that was not significantly different from 0°). The ME, however, a ~2.1° reduction in absolute pointing error after spatially congruent exposure, occurred regardless of the exposure protocol. A similar pattern was found in Experiment 2 where the displacement of the light was not fixed at either 0° or 13.5°, but slightly varied around these means, thus, suggesting that the VAE and ME take the average audiovisual displacement into account, and not its variance. Finally, Experiment 3 was an auditory‐only control condition where during exposure sounds were presented in isolation, so without lights and audiovisual displacement. The data showed that in this case there was no shift (VAE) and no reduction in variance (ME) between pretest and posttest.

How to account for the finding that the VAE depends on the rate at which exposure stimuli are presented, while the ME is immune? The authors argued that the VAE and ME dissociate because different neural structures underlie these effects. Previous research with patient groups indeed suggests that the VAE and ME might be mediated by dissociable mechanisms (Passamonti et al., 2009). The authors reported that hemianopic patients with lesions of striate cortex did not have a VAE in their blind field (while the ME was spared), whereas neglect patients with lesions in temporoparietal cortex had a normal VAE and ME in their neglected field. The VAE was thus again more vulnerable than the ME, and this dissociation thus may suggests that the VAE requires an intact striate cortex, whereas the ME relies on different neural circuits than the ones that are causing neglect or hemianopia.

At this stage, however, it remains to be further tested why the temporal pattern of audiovisual stimulation (2Hz versus 10 Hz) affects the VAE and not the ME. One viable option why the VAE diminishes at 10 Hz is that the neural integration of spatial attributes of a sound and light takes time, and that the presentation rate of 10 Hz is too fast for spatial integration to occur. With electroencephalography (EEG), it has been indeed reported that a ventriloquist effect becomes detectable only around 260 ms postonset (Bonath et al., 2007; Stekelenburg et al., 2004). For sounds presented in sequence, a similar upper limit of around 4 Hz has been reported for tasks in which participants had to judge audiovisual temporal synchrony (Fujisaki & Nishida, 2005), so that at stimulation rates above 4 Hz, participants are no longer able to judge whether sounds and lights are synchronous or asynchronous.

To the best of our knowledge, however, this critical 4 Hz range has not been tested for the VE and the VAE. This knowledge is nevertheless critical because it allows one to more specifically pinpoint the frequency at which the VAE and VE fall apart. This knowledge is crucial for alignment with other research on multisensory integration. Conceivably, one can examine the VE (but also the VAE) in task as described in Vroomen and Stekelenburg (2014). They used a two‐alternative forced‐choice task (2AFC) in which participants were presented a static and a left/right alternating sequence of sounds at a rate of 2 Hz. The participants' task was to decide which of the two sequences contained the alternating sequence, first or second? Results showed that when the two sound sequences were accompanied with left/right alternating flashes, discrimination of static sound from alternating sounds became much more difficult because the alternating lights made the static sounds appear to alternate as well. The critical question for future research would be at which frequency this VE breaks down: We would put our money on 4 Hz.

But why then did the presentation rate at 10 Hz not affect the ME? The authors argued that the selective reduction in the VAE was due to a specific temporal limitation of the neural circuitry required for audiovisual recalibration and not due to a general impairment of multisensory integration or other unspecific effects. However, a double dissociation between the VAE and the ME (i.e., sparing of VAE while the ME is harmed) has not been found. A straightforward option that remains, in our view, on the table is that the VAE is just more vulnerable than the ME possibly because it declines faster, is easier to erase, requires more time to build up, is just more difficult to measure, and so forth.

More importantly, what is also lacking at this stage is a visual‐only control condition for the ME that accounts for the fact that participants in due course of the experiment become aware of the experimental set‐up. During audiovisual exposure, participants become aware of the set‐up because the lights provide feedback on where the hidden speakers are located. This higher‐order knowledge about speaker locations (e.g., that there are only 6, in congruent conditions 3 on the left, and 3 on the right; in displaced conditions 2 speakers on the left, 4 on the right; that there is no speaker in the middle; that speakers are separated by 13.5° and range between −22° to +22°; etcetera) can be deduced from the lights during low‐ *and* high‐frequency stimulation, but is absent in the sound‐only condition where there was no ME. Possibly, then, the ME survives because the lights provide feedback about the speaker locations in the set‐up. Therefore, various alternatives need to be checked before heavy theoretical or neural claims are made that dissociate the VAE from the ME.

## CONFLICTS OF INTEREST

Authors declare that there are no relevant financial or non‐financial competing interests.

### Peer Review

The peer review history for this article is available at https://publons.com/publon/10.1111/ejn.15001.
